# Effects of Calcined Coal Gangue and Carbide Slag on the Properties of Cement Paste and Mortar

**DOI:** 10.3390/ma18102242

**Published:** 2025-05-12

**Authors:** Yudong Luo, Yonghong Miao, Peng Wang, Panpan Gai, Jingwei Yang, Guiyu Zhang

**Affiliations:** 1 Faculty of Civil Engineering and Mechanics, Jiangsu University, Zhenjiang 212013, China; 2232323004@stmail.ujs.edu.cn (Y.L.); yhmiao@ujs.edu.cn (Y.M.); upeswp@ujs.edu.cn (P.W.); gai@ujs.edu.cn (P.G.); 2 Department of Civil and Environmental Engineering, Seoul National University, Seoul 08826, Republic of Korea; snuyangjw@snu.ac.kr

**Keywords:** carbide slag, microstructure, calcined coal gangue, hydration reaction, sustainability

## Abstract

When using supplementary cementitious materials to replace cement partially, the carbon emissions of cement products can be reduced, but it often leads to reduced strength. This study explores the application potential of carbide slag (CS) and calcined coal gangue (CCG), byproducts of acetylene production, to partially replace cement. The effects of these two materials on the macroscopic properties and microstructure of cement-based materials were analyzed through systematic experiments. The compressive strength, ultrasonic pulse velocity, and electrical resistivity test results showed that replacing 20% of cement with CCG did not cause significant changes in the test results of the specimens. An X-ray diffraction (XRD) analysis showed that these two materials can produce additional hydration products. Scanning electron microscopy images (SEM) further revealed that CCG produces hydration products to fill microscopic pores. Thermogravimetric analysis (TG) results after 28 days showed that with the addition of supplementary cementitious materials, calcium hydroxide (CH) in CS reacts with CCG, resulting in the consumption of CS. Finally, the environmental impact of CS and CCG was assessed. It was found that CS is more favorable for reducing carbon emissions compared to CCG. However, when considering the effect of cement replacement on compressive strength, combining these two materials is more advantageous for sustainable development. Overall, the use of CS and CCG demonstrated good performance in promoting sustainable development.

## 1. Introduction

Human activities have significantly increased the amount of greenhouse gases in the atmosphere [1]. Cement is an important component of building materials, and its production process is one of the main sources of greenhouse gases. The cement industry releases 5–8% of global anthropogenic carbon dioxide emissions [2,3,4,5,6,7,8]. To reduce carbon emissions from the cement industry, researchers have used supplementary cementitious materials (SCMs) with volcanic ash properties to replace cement and reduce cement content, thereby reducing carbon dioxide emissions and energy consumption [9].

Calcium carbide slag (CS) is an industrial by-product of acetylene production. Acetylene (C_2_H_2_) is usually produced by the reaction of calcium carbide with water. This process produces a large amount of CS [10,11,12]. The reaction process is as follows:

(1) $$CaC_{2}+2H_{2}O\to \;C_{2}H_{2}+Ca(OH{)}_{2}$$

The main component of solid waste carbide slag is calcium hydroxide (CH). If it is not handled properly, it can easily lead to problems such as soil salinization and groundwater pollution [13]. Its application in cement and concrete is mainly due to its chemical composition, calcium hydroxide (CH): CS can act as an alkali activator in the cement hydration process, reacting with other cementitious materials to generate additional C-S-H and other hydrated compounds. These additional C-S-H fill the pores in the mixture by promoting pore refinement and thickening of the cementitious matrix, thereby improving the density and durability of the material.

China’s coal production accounts for about 50% of the world’s coal production [14]. As one of the main solid wastes in the coal mining process, coal gangue (CG) accounts for 10% to 20% of coal production [15,16]. From 2010 to 2020, China has produced a total of about 6 billion tons of CG [17]. The accumulation of CG not only occupies a large amount of land resources, but also the heavy metal elements it contains may seep into groundwater, posing a potential threat to the ecosystem and human health [18]. The total amount of CG in China is still increasing year by year. In China, the utilization rate of CG (about 60%) is much lower than that of developed countries (greater than 90%). The annual output exceeds the utilization, indicating that the utilization rate of CG still needs to be improved [19]. The annual output of CG is much higher than the utilization, indicating that the utilization rate of CG still needs to be improved [19]. CG itself is difficult to react with substances in cement, but the calcined coal gangue (CCG) obtained after fine grinding and heat treatment is active. During the calcination heat treatment process, kaolin removes hydroxyl groups to form metakaolin, thereby increasing the reaction activity [20,21]. Most previous studies have focused on replacing aggregates in concrete with CG [22,23,24]. At present, some researchers have found the potential of CG or CCG to replace cement [25,26,27]. However, research on the interaction between CCG and other SCMs in cementitious systems still needs to be supplemented.

Because of previous studies’ shortcomings, this paper’s innovation is summarized as follows: First, previous studies have mostly focused on using CG as a substitute for aggregate, and the research on CG replacing cement after activation treatment needs to be further supplemented. Second, existing studies are mostly based on macroscopic physical properties, and the research on the microscopic properties of CCG and its influence on the performance of cement-based materials still needs to be strengthened. Finally, this study explores the effect of mixing CS and CCG and analyzes the possible interactions and effects of this mixed cementitious material.

The variables considered in this study are the dosage of CS and CCG. Experimental studies on mortar specimens and neat paste samples include compressive strength, ultrasonic pulse velocity (UPV), electrical resistivity (ER), X-ray diffraction (XRD), thermogravimetric analysis, and scanning electron microscopy (SEM). Based on these experimental results, the macroscopic properties and microstructure of the samples and the correlation between the experimental results are analyzed.

## 2. Materials and Methods

### 2.1. Materials

**Figure 1:**
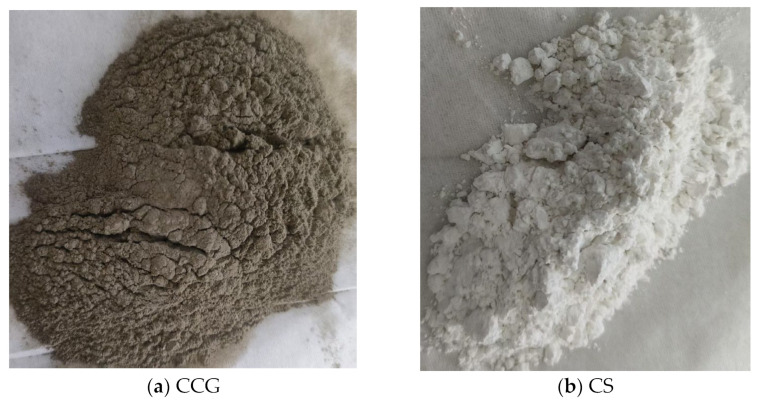
Photos of both CS and CCG.

This study used 42.5-grade ordinary Portland cement (OPC), CS, and CG as raw materials to prepare mortar specimens and paste samples. Figure 1 presents the original images of CCG and CS. The obtained CG and CS were subjected to a sieving treatment. This was aimed at regulating and optimizing their particle size distributions to make them closer to that of cement. OPC was purchased from Jiangsu Helin Cement Co., Ltd., Zhengjiang, China, and its specific surface area is 3858 cm^2^/g. CG was sourced from Yangcheng Yongqing Coal Gangue Processing Plant, Yangcheng, China. The CS was from Gongyi Yuanheng Water Purification Materials Plant, Gongyi, China. Experimental standard sand and laboratory tap water were used.

**Figure 2:**
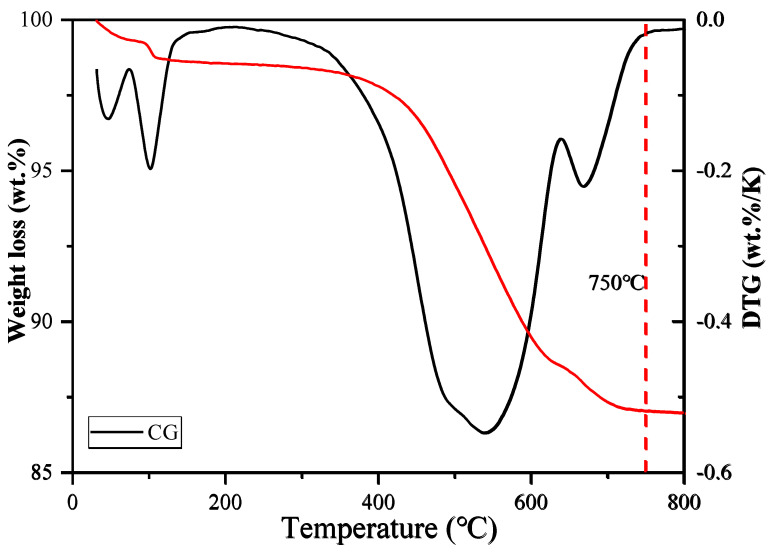
Weight loss (%) registered by thermal analysis data: thermogravimetry (left) and differential thermogravimetry (right).

In order to determine the appropriate calcination temperature, some coal gangue (CG) samples were first subjected to thermogravimetric analysis (TGA). The experimental results showed (see Figure 2) that the sample completed most weight loss processes at 750 °C, so 750 °C was selected as the calcination temperature. During the calcination process, the coal gangue sample was placed in a muffle furnace, heated to 750 °C at a heating rate of 10 °C/min, and kept at this temperature for 2 h. Subsequently, the sample was naturally cooled to room temperature, and finally, the calcined coal gangue (CCG) was obtained.

**Figure 3:**
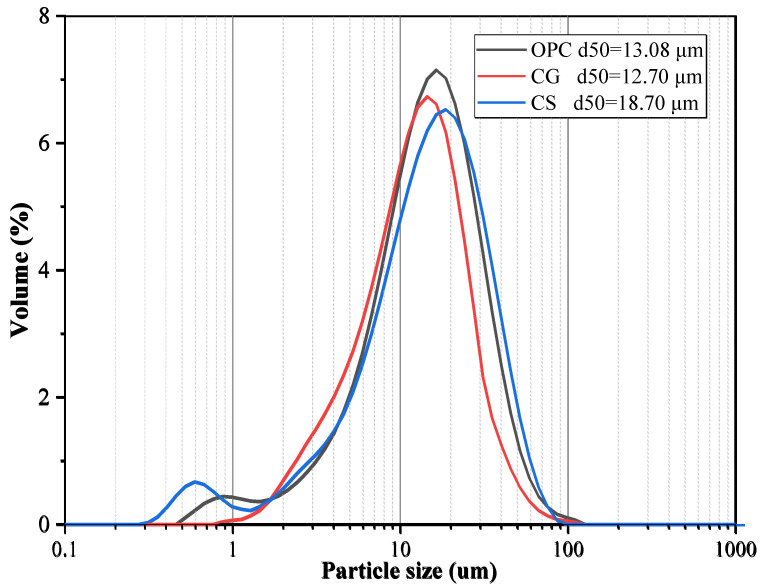
Particle size distributions of OPC, CG, and CS.

**Figure 4:**
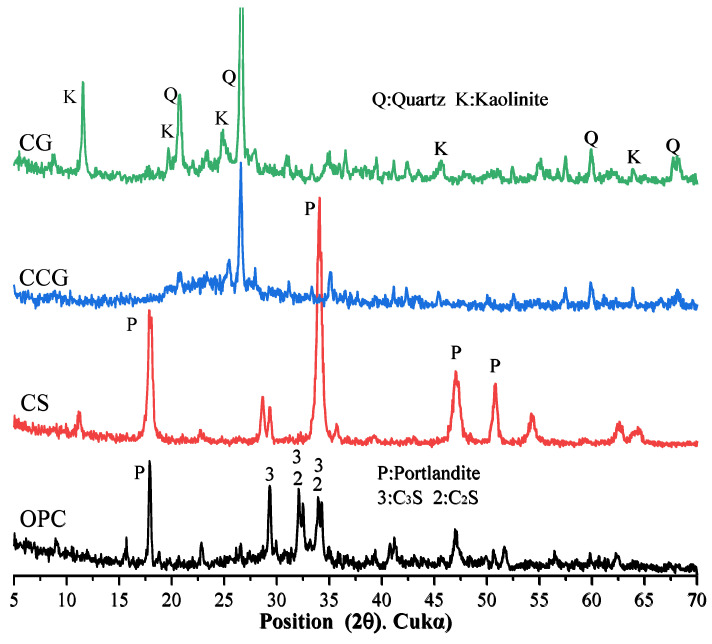
XRD patterns of OPC, CS, CG, and CCG.

The OPC, CG, and CS oxide contents were determined by X-ray fluorescence (XRF), and the results are shown in Table 1. The particle size distribution (PSD) curves of OPC, CCG, and CS, obtained using a particle size distribution analyzer, are shown in Figure 3. According to the ASTM C188 standard [28], OPC, CG, and CS densities are 3.14, 2.56, and 2.53 g/cm^3^, respectively. The XRD test results of OPC, CCG, CG, and CS are shown in Figure 4. The results show that the main mineral components of CG are quartz and kaolin. After calcination, kaolin’s characteristic peak (K) is significantly reduced or even disappears, indicating that calcination converts kaolin in CG into metakaolin, thereby enhancing its activity [25,29].

### 2.2. Mixing Ratio and Sample Preparation

Cement replacement was performed by using CS and CCG in single, double, and no addition combinations, as detailed in Table 2 for each mortar mix. To ensure the optimal performance of cement products, the proportion of cement replacement by industrial waste SCM should be limited to within 40% [30]. Mortar specimens (40 mm × 40 mm × 160 mm) and cement paste samples (40 mm × 40 mm × 40 mm) were prepared accordingly. After the initial 24-h curing under standard conditions, the samples were demolded and transferred to a standard curing room for further curing.

### 2.3. Experimental Methodology

#### 2.3.1. Workability Test

According to ASTM C1437-01 (2001) [31], the fluidity of fresh mortar is assessed by the following steps: Prepare the mortar sample using a standard cone mold, vibrating table, and measuring scale. Strike off the mortar level with the top of the mold. Lift the mold one minute after mixing, immediately drop the flow table 25 times, and measure the diameter of the mortar at four lines on the top of the table with a caliper, recording each diameter to the nearest tenth of a scale division.

#### 2.3.2. Compressive Strength Test

The test was conducted using a fully automatic pressure testing machine (DY-208JC, Wuxi Orient Instrument Making Technology Co., Ltd., Wuxi, China). The compressive strength loading rate was 2400 N/s. The flexural strength of the mortar specimens was tested using 40 mm × 40 mm × 160 mm specimens, and the broken specimens were further tested for compressive strength. The specimens were cured in a standard curing box for 1 day, 3 days, 7 days, and 28 days, respectively. The average strength of the three mortar specimens was taken as a result.

#### 2.3.3. Electrical Resistivity Test

The ER test was carried out using a non-destructive resistivity tester. For each mortar specimen with each ratio, three sizes of 40 mm × 40 mm × 160 mm were cast, and the ER was measured at 1 day, 3 days, 7 days, and 28 days. Before the test, the surface of the specimen was wiped clean with a towel, and the resistivity values of the three specimens were tested. Under the condition of meeting the extreme difference, the average value was taken as the test result.

#### 2.3.4. Ultrasonic Pulse Velocity

The UPV test was performed on the mortar specimens cured for 1 day, 3 days, 7 days, and 28 days using a non-metallic ultrasonic detection analyzer (ZT805, Nanjing Zhongtuo Technology Co., Ltd., Nanjing, China). The size of the tested mortar specimens was 40 mm × 40 mm × 160. The UPV values of the three specimens were tested, and the average value was taken as the test result to meet the extreme difference.

#### 2.3.5. Microscale Tests

XRD and TG analyses were performed on the slurry samples on day 1 and day 28 of curing, respectively. Small pieces of the sample were removed from the slurry’s inside, soaked in isopropanol for at least 7 days to stop hydration, and then dried in a vacuum dryer for 24.0 h. Subsequently, the sample was ground into powder and passed through a 45 μm sieve.

The crystal phase composition of the hydration product of the pure slurry sample was analyzed by X-ray diffractometer (XRD6100, Shimadzu Corporation, Kyoto, Japan). The Cu Kα radiation current was 40 mA, the voltage was 40 kV, the 2θ range was 5–70°, and the scanning speed was 5°/min.

A thermogravimetric analyzer (NETZSCH, Selb, Germany) was used in a nitrogen (N_2_) environment, and the temperature was increased from room temperature to 800 °C at a heating rate of 15 °C/min. Thermogravimetric analysis (TGA) and its first-order derivative (DTG) determined the hydration products and CH content in each compound.

#### 2.3.6. SEM Teste

The microstructure of the pure slurry sample was analyzed by field emission scanning electron microscopy (TESCAN, Brno, Czech Republic). Small pieces of the pure slurry sample that had been cured for 28 days were soaked in isopropanol for 7 days to stop hydration. After hydration stopped, they were dried in an oven at 80 °C for 24 h. A thin layer of gold was then plated to obtain good conductivity.

## 3. Results

### 3.1. Workability

**Figure 5:**
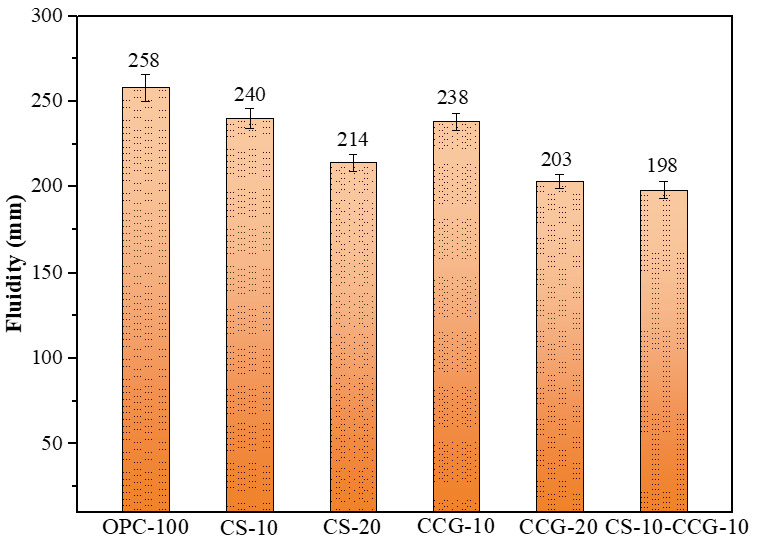
Fluidity results.

As shown in Figure 5, the fluidity of the paste decreases from 258 mm to 214 mm as the CS replacement ratio increases, exhibiting a downward trend. This is attributed to the high specific surface area of CS, which adsorbs free water and reduces the effective water–binder ratio, thereby decreasing fluidity. After partial replacement of cement with CCG, the fluidity of cement mortar is significantly reduced. This can be ascribed to the fact that CCG still contains trace amounts of coal and organic materials, which can absorb 30–65% of water by their own weight, leading to a lower actual water–binder ratio and reduced fluidity [32]. Additionally, after calcination, kaolinite is transformed into metakaolinite, resulting in a more porous structure and higher water absorption, which further reduces the fluidity of the mortar [33]. Figure 5 shows that using CCG reduces the mortar’s fluidity a bit more. Hence, to match OPC’s performance, groups with CCG need more water-reducing admixture.

### 3.2. Compressive Strength

**Figure 6:**
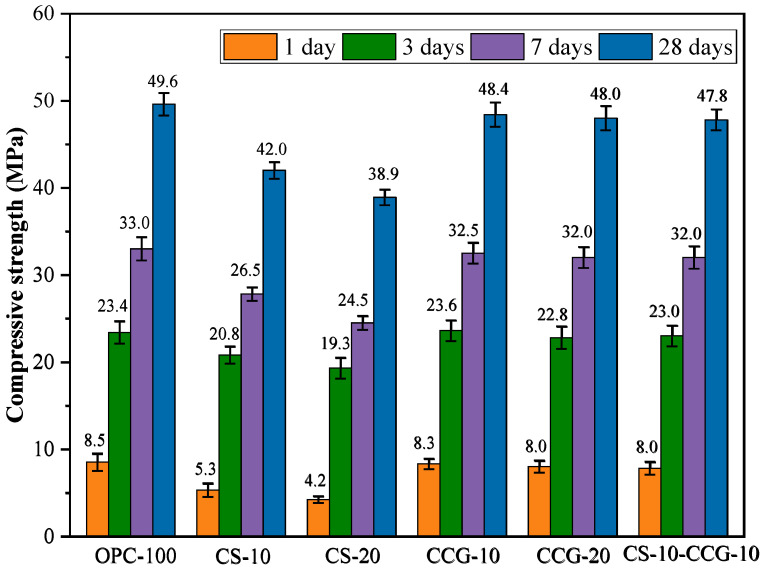
Compressive strength results.

Figure 6 shows the compressive strength test results of the mortar specimens at 1 day, 3 days, 7 days, and 28 days. The experimental results show that compressive strength increases with the extension of the curing time. This is because the hydration reaction continues, the number of hydration products increases, and the cement becomes denser.

By comparing the compressive strength results of each group, it can be found that the compressive strength of the mortar specimens does not decrease significantly with the increase in CCG substitution. The main reason for this phenomenon is that CCG’s amorphous phase metakaolinite (AS_2_) has a high reactivity [34]. AS_2_ can react with CH produced by cement hydration in the early hydration stage. The reaction equations are Equations (2) and (3) [34].

(2) $$AS_{2}+6CH+9H\to 2C-S-H+C_{4}AH_{13}$$

(3) $$AS_{2}+3CH+6H\to C-S-H+C_{4}AH_{13}$$

where CH is portlandite, $AS_{2}$ is amorphous phase metakaolinite, and H is water. The reaction produces additional hydration products, such as C-S-H and C-S-A-H, which can promote strength development.

In addition, it can be observed that the use of CS to replace cement will reduce the compressive strength of the CS experimental group. At 1, 3, 7, and 28 days, they decreased by 50.59%, 17.5%, 25.7%, and 21.5%. This is mainly because the main substance in CS is CH. When cement is partially replaced, the number of hydration products decreases, resulting in a decrease in strength. This can be attributed to the dilution effect of CCS, which leads to a reduction in strength [35]. The compressive strength of the 10-CS-10-CCG group reached a level comparable to that of the control group. This is mainly because the CH in CS can react with AS_2_ in CCG to produce (2) and (3). The C-S-H and C-S-A-H produced by the reaction can fill the capillary tubes in concrete [36,37]. Therefore, mixing CS and CCG can effectively promote the development of strength.

### 3.3. UPV

**Figure 7:**
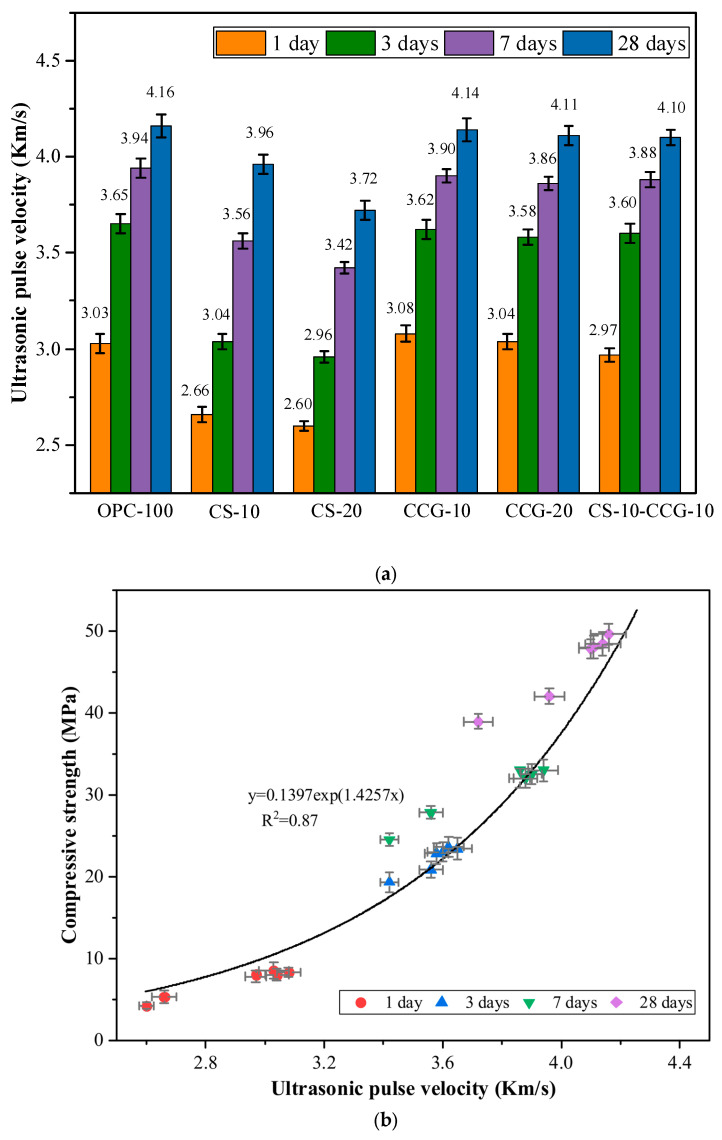
(**a**) UPV test results. (**b**) Relationship between compressive strength and UPV.

Figure 7a shows the UPV test results of each group of mortar specimens. The UPV test results of OPC-100 (control group) at 1, 3, 7, and 28 days were 3.03, 3.65, 3.94, and 4.16 km/s, respectively. The main reason for the rapid increase in UPV at 1 day was the rapid hydration reaction of C_3_S and C_2_S in cement to form CH and C-S-H [38]. However, after 3 days of curing, the cement hydration reaction and the increase in UPV test results slowed down significantly [35].

By comparing the UPV data of each group, it can be seen that the UPV values of the groups added with CCG have not decreased significantly compared with the control group. This is because, in the early stage of hydration, the high reactivity of CCG can react with CH produced by the hydration of cement clinkers. The hydration products generated by the reaction can fill the internal pores and promote the growth of UPV. In addition, the literature pointed out that the crystallinity of CG will be significantly reduced after calcination [29]. The crystallinity is related to the arrangement of molecular chains. The higher the crystallinity, the more orderly the molecular arrangement, which may make the reaction more difficult. The crystallinity of CG after calcination is reduced so that CG can obtain higher reactivity.

The test results of CS-10 and CS-20 mortar specimens were smaller than those of the control group. For example, the value of the CS-20 group was 14.20% lower than that of the control group at 1 day. It was 10.58% lower than that of the control group at 28 days. This is because CS contains a large amount of CH, and as the curing age increases, its reactants are limited, and more hydration products cannot be generated. When CS and CCG are mixed, the CH in CS can react with the silicon aluminum oxide in CCG, promoting the growth of UPV value.

Factors that affect the UPV value, such as the amount of hydration products and pore size, also have a crucial impact on the compressive strength [39]. UPV can be used to evaluate and predict the compressive strength of concrete in practical engineering. Previous studies have revealed a strong correlation between UPV and compressive strength, and there is an exponential correlation between the two [39,40]. The correlation analysis between UPV and compressive strength of the mixed mortar in this study is shown in Figure 7b, with R^2^ = 0.87.

### 3.4. Electrical Resistivity

**Figure 8:**
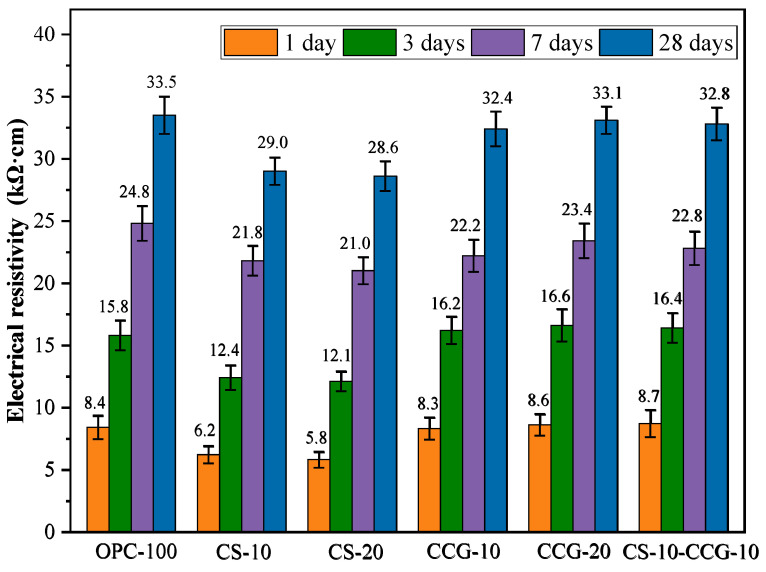
Electrical resistivity of blended pastes at 1, 3, 7, and 28 days.

The ER is an important factor in evaluating the durability of cement-based mixtures. The larger the ER of the mortar, the lower the corrosion rate of the steel bars inside it. Figure 8 shows the ER of the mortar specimens at various ratios at 1, 3, 7, and 28 days. The ER value increases with age. This is because as the hydration reaction proceeds, the pores in the mortar are filled, and the interior becomes denser.

At all test time points, the ER values of the mortar containing CCG were comparable to those of the control group. This indicates that the use of CCG to partially replace cement will not cause a significant decrease in the ER value. This indicates that the density and durability of the cement system are enhanced in the presence of CCG, mainly because CCG can participate in the reaction to produce C-S-H and C-A-S-H. The ER values of the CS-20 group at 1, 3, 7, and 28 days were significantly lower than those of the control group. For example, the ER values of the CS-20 group at 1, 3, 7, and 28 days decreased by 30.95%, 23.41%, 15.30%, and 14.63%, respectively, compared with the control group. The volcanic ash effect of CS and CCG is manifested in that SiO_2_ and Al_2_O_3_ react with CS and cement CH in the cementitious system to generate cementitious materials such as C-S-H and C-S-A-H, which improves the density and volume stability of the matrix and makes the penetration of chloride ions more difficult.

Numerous studies have investigated the relationship between the ER value of concrete and its resistance to chloride ion corrosion [41]. The findings indicate a correlation between the ER value and the concrete’s ability to resist chloride ion penetration, suggesting that the ER test can effectively evaluate this resistance. It has been noted that if the ER value exceeds 20,000 Ωcm, the likelihood of corrosion is considered low [42]. Based on the test results presented in Figure 8, we can conclude that all test groups show a low probability of corrosion after 7 days of curing.

### 3.5. X-Ray Diffraction Analysis

**Figure 9:**
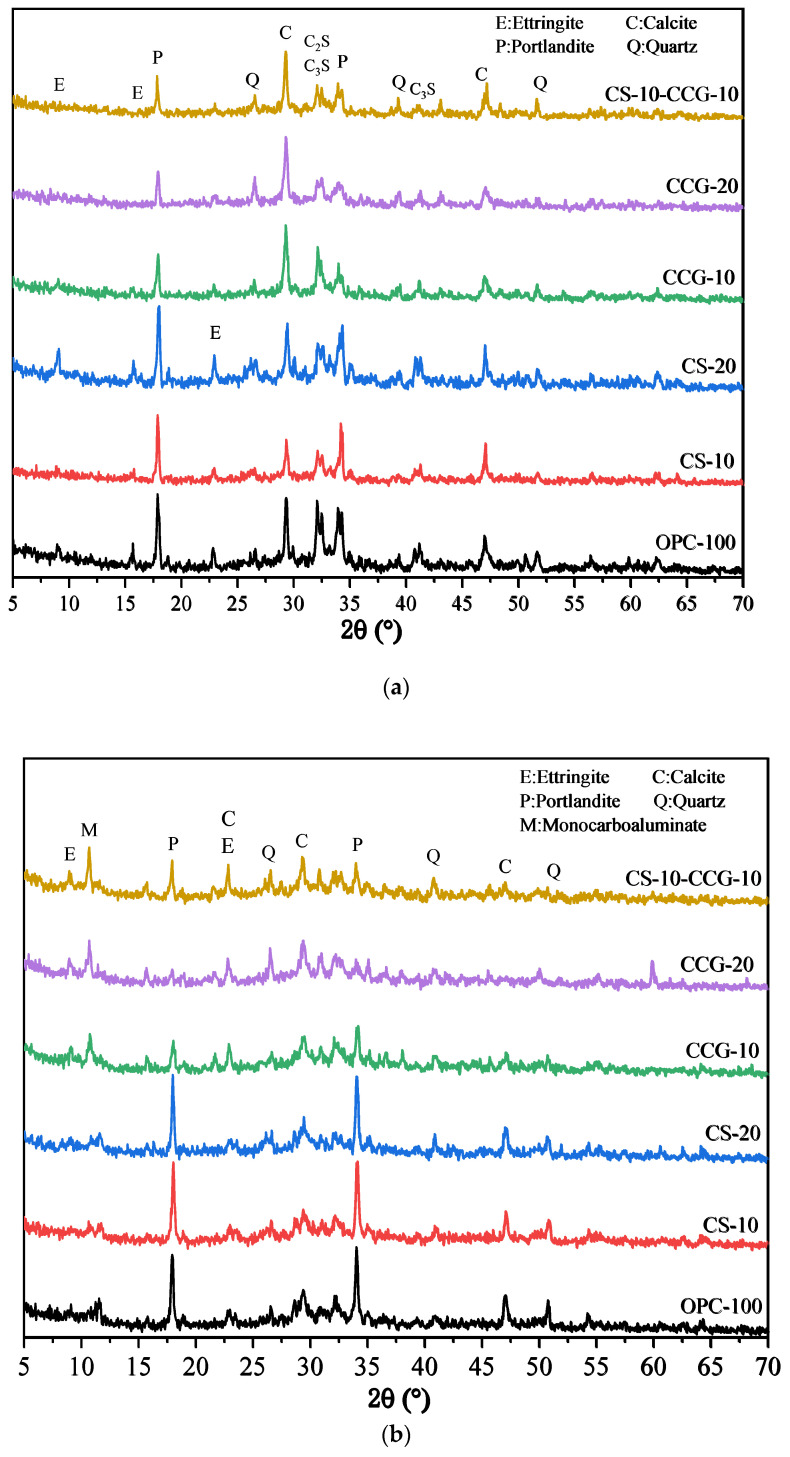
XRD patterns of the blended pastes at (**a**) 1 day and (**b**) 28 days.

Figure 9a,b show the XRD test results of all samples at 1 day and 28 days. The relevant characteristic peaks are summarized in Table 3. It can be observed from the figure that after the addition of the two cementitious materials, the main types of products of the hydration reaction did not change significantly. In addition, the peak of calcium carbonate (2θ = 46.5°) was detected in the cement system because the cement used contained limestone [43]. It can be observed in Figure 9a that the peaks of C_3_S and C_2_S are higher than those in Figure 9b, which is due to the fact that the C_3_S and C_2_S in the early hydration reaction are not yet fully formed. When the reaction proceeds to 28 days, the peaks of C_3_S and C_2_S drop significantly because, after 28 days of hydration, the C_3_S and C_2_S in the system react to generate other hydration products.

By comparing Figure 9a and Figure 9b, it can be found that the P peak of the group with CS addition is very obvious, which is mainly because the CH in CS is not fully reacted and is detected. In addition, it can be found that the M peak at 11.7° of the component doped with CCG from the 1st day to the 28th day of the reaction is more obvious than that of other groups, while the peak of calcium carbonate decreases more significantly. This is because the calcium carbonate in the mixture reacts with aluminate to generate M. The reaction equation for the generation of M is shown in Equation (4) [35].

(4) $$3CH+CC+9H+A\to C_{3}A\cdot CC\cdot H_{12}$$

where CH is portlandite, CC is calcium carbonate, H is water, and A is the aluminum phase in cement.

By analyzing this equation and the principle of chemical reaction, this is because CCG contains more Al elements, which can provide aluminum sources for the reaction, thereby promoting the generation of M. It can also be found from Figure 9b that the CH peak intensity at 18° in the CCG-doped group is significantly reduced, which indicates that CCG is a raw material with high chemical activity and is easy to react with CH generated by cement hydration.

### 3.6. Thermogravimetry

**Figure 10:**
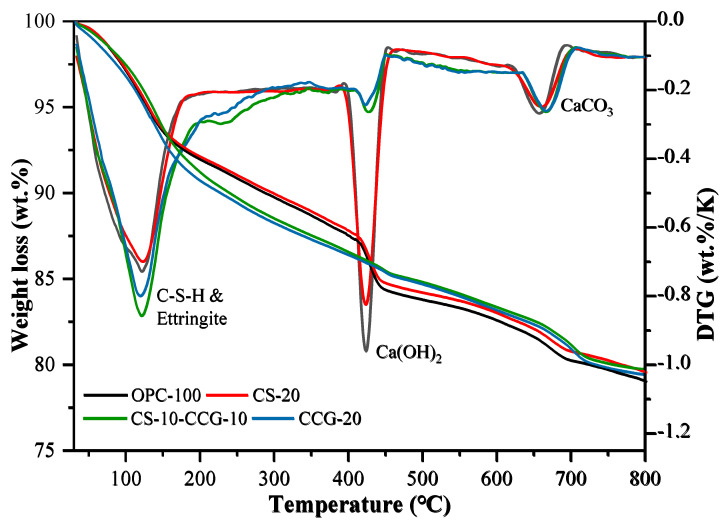
Weight loss (%) registered by thermal analysis data: thermogravimetry (left) and differential thermogravimetry (right) of the pastes studied at the ages of 28 days.

Figure 10 shows the TG and DTG curves of OPC-100, CCG-20, CS-20, and CS-10-CCG-10 samples. The following three different peaks were observed in all samples, each corresponding to a different material decomposition process: 1. The low-temperature peak (105–300 °C) is due to the dehydration of C-S-H, C-S-A-H, AFt, and M [44,45]. 2. The medium temperature peak (400–500 °C) is related to the decomposition of CH [46,47]. 3. The high temperature peak (500–800 °C) is related to the decomposition of calcium carbonate [48,49]. Table 4 shows the percentage of mass loss in these four stages.

From the calculation results in Table 4, it can be seen that the addition of CCG powder reduces the content of CH. This result is consistent with the change in the CH peak intensity observed in the XRD image of the component added to CCG. There are two main reasons for this fact. First, with the replacement of cement with CCG, the content of active clinkers will decrease in the same proportion, and the CH produced by clinker hydration will also decrease. On the other hand, CCG reacts with CH, and Si^4+^ and Al^3+^ in CCG react with CH to form C-S-H and C-A-S-H, which is also the reason for the higher compressive strength in the later period [34]. In addition, it can be found that the CH content of the CS-20 group is higher than that of other groups, and it decomposes less in the low-temperature range. This is mainly because the CH contained in the reactant CS decomposes at high temperatures, and there are fewer reactive silicon and aluminum substances. The calcium carbonate that decomposed in the high-temperature peak mainly comes from the limestone in the cement.

### 3.7. SEM

**Figure 11:**
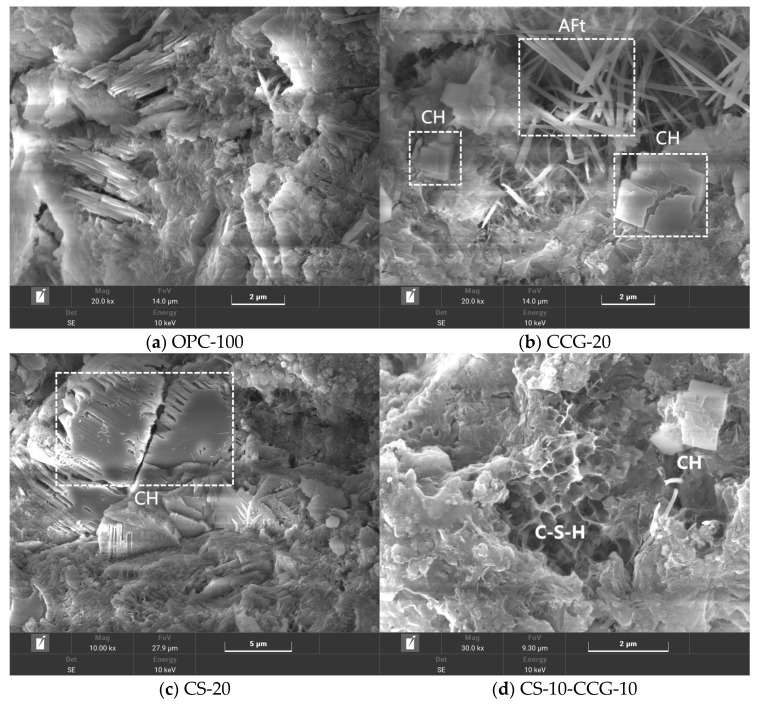
SEM images at 28 d of OPC-100, CCG-20, CS-20, and CS-10-CCG-10 pastes.

Figure 11 shows the microstructure of OPC-100, CS-20, CCG-20, and CS-10-CCG-10 paste samples at 28 days. In OPC-100, common hydration products such as needle-shaped AFt, as well as CH crystals of different morphologies (e.g., flat, prismatic, and long crystals, or plate-like and hexagonal structures), can be observed. These hydration products fill the pores inside the cement and are the main reason for the hardening and strength development of cement.

Figure 11b–d show the SEM images of CS-20, CCG-20, and CS-10-CCG-10 samples, respectively. By comparing these three images, it can be found that there are a large number of CH crystals in CS-20, which indicates that the use of CS to replace cement may lead to a shortage of reactants, and the excess CH in CS exists in the cement cementitious system in the form of crystals. In contrast, when CCG partially replaces cement (as seen in the CCG-20 group), needle-like AFt can be observed within the samples. This can be attributed to the chemical reactions between the reactive substances present in CCG (such as SiO_2_ and Al_2_O_2_) and CH in CS and cement. This is consistent with the intensity pattern shown by the AFt chemical peak in XRD tests.

### 3.8. Carbon Emission Analysis

In recent years, the continuous growth of global carbon emissions has become one of the key factors driving climate change [34,35,36]. The greenhouse effect caused by carbon emissions has led to significant changes in the global climate, which in turn has caused a series of environmental problems, including frequent extreme weather events and rising sea levels [37]. These problems not only pose a serious threat to the stability of the ecosystem but also pose a huge challenge to the sustainable development of human society. In order to achieve the goal of sustainable development and ensure the healthy development of ecology and society, it has become particularly urgent to take effective carbon emission reduction measures. Therefore, this study calculated the carbon dioxide emissions generated by the production of one cubic meter of mortar. In the calculation process, only the carbon dioxide emissions under the experimental mix ratio were considered, and the carbon emissions generated during transportation and mixing were ignored. Table 5 shows the material mass required to produce 1 cubic meter of experimental mortar, Table 6 shows the carbon dioxide emission factor (CO_2_ kg/kg) per unit mass of the raw materials used in this experiment, and Table 7 shows the calculation results of carbon emissions. Equation (5) limits the total volume of each mortar component (OPC, CS, CCG, sand, and water) to 1 m^3^.

(5) $$\sum_{i=1}^{n}\frac{m_{i}}{\rho_{i}}=1$$

where $\rho_{i}$ represents the raw material density; $m_{i}$ is the raw material unit volume density (kg/m^3^), n = 5; and i represents each component in the raw material mixture (OPC, CS, CCG, sand, and water).

According to Equation (6):

(6) $$CO_{2}P=\sum_{1}^{n}CO_{2}m_{i}$$

The CO_2_ emissions and energy consumption per unit volume were calculated, where “CO_2_P” is the CO_2_ emissions per cubic meter of mortar and mi is the mass of each raw material per unit mass (tonnes) of mortar mix. “CO_2_” is the CO_2_ emission factor of the raw material (CO_2_ kg/kg).

**Table 6:** Carbon footprint of raw materials used in this investigation [50,51,52].

Raw Materials	OPC	CS	CCG	Sand	Water
GWP(CO_2_ kg/kg)	0.86	0	0.079	0.0026	0.000196

**Table 7:** Carbon consumption of raw materials used in this investigation results.

Group	OPC-100	CS-10	CS-20	CCG-10	CCG-20	CS-10-CCG-10
CO_2_ (kg)	545	488	432	493	442	437

The calculation results in Table 7 show that as the proportion of cement replaced by these two materials increases, the carbon dioxide emissions per unit volume of mortar decrease significantly. The largest reduction is in the CS-20 group, which is 20.66%. This is because CS is regarded as an industrial by-product, so its carbon emissions are regarded as zero.

**Figure 12:**
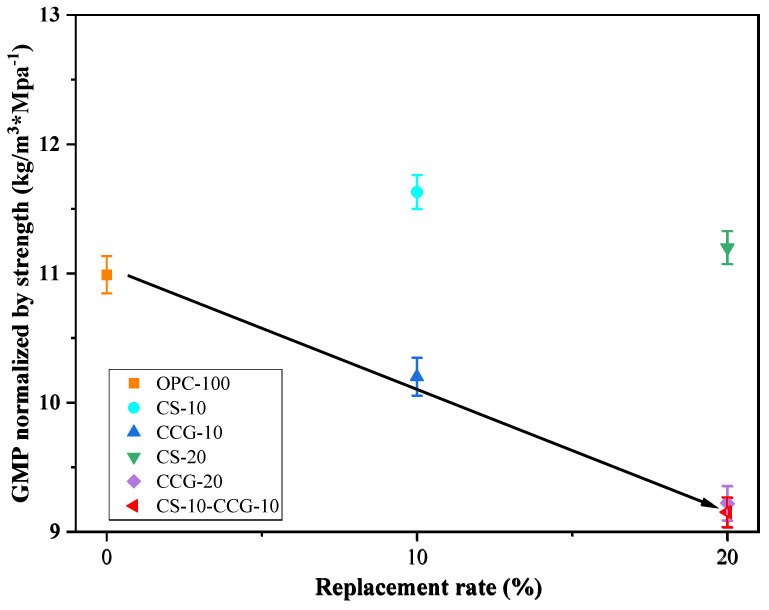
Strength-normalized CO_2_ emissions versus the SCM’s replacement rate.

In actual engineering applications, while considering the reduction of CO_2_ emissions, we should also consider the changes in cement mix proportions and the changes in cement product strength that will result. According to Equation (7) [50], the 28-day compressive strength of each group of sample mortar is normalized to CO_2_ emissions.

(7) $$ECO_{2}=\frac{CO_{2}P}{F_{28}}$$

where $ECO_{2}$ is the CO_2_ emission per unit compressive strength of 28 days (kg·CO_2_/MPa); $F_{28}$ is the compressive strength of the mortar sample after 28 days of curing; and $CO_{2}P$ is the calculated CO_2_ emission per unit volume. From the results in Figure 12, it can be found that when considering compressive strength, the use of CS does not have a good effect compared to the use of CS. However, when the two are mixed, the carbon dioxide emissions per unit of compressive strength are reduced to a greater extent. The mixed group shows the best result in these data, i.e., 9.15 kg·CO_2_/MPa.

## 4. Conclusions

This paper aims to investigate the use of CCG and CS as cement-based supplementary materials to improve the sustainability of construction practices. Specifically, this study partially replaced cement with CCG or CS and focused on the compressive strength, UPV, ER, XRD, TGA, SEM, and carbon emissions of the samples. The conclusions drawn from the experiments are as follows:

1. The compressive strength, UPV, and ER test results show that when the cement part is replaced by CS, the compressive strength, UPV, and ER of the mortar specimens decrease. However, when the cement part is replaced by CCG, the compressive strength, UPV, and ER of the mortar specimens will not decrease significantly. This is mainly related to the high reactivity of CCG.
2. The XRD experimental results show that when part of the cement is replaced by CCG, the silicon and aluminum components in CCG will react with the cement hydration to produce CH and be consumed. In addition, the XRD experiment also shows that when the cement is replaced by CS, the CH content in the sample will increase.
3. The TG experimental results show that when part of the cement is replaced by CCG, the CH content in the sample decreases, and the content of other hydration products increases. This is mainly because after CCG is used to replace cement, the CH in the sample will react with CCG and be consumed.
4. SEM images show that the samples with CCG added produce hydration products to fill the pores, and these additional hydration products can improve the interface between CCG and cement, which is attributed to the reactivity of CCG. However, there is a large amount of CH inside the samples with CS added.
5. The analysis of carbon emissions indicates that the use of both materials is beneficial for reducing carbon emissions. Furthermore, when considering the relationship between carbon emissions and 28-day compressive strength, the results of using CCG alone or mixing CCG with CS are superior to using CS alone.

This paper conducted a feasibility study on the utilization of CS and CCG, which provides a promising solution for treating CG produced by coal and CS produced as a byproduct of acetylene production and reducing the environmental burden of cement products without affecting the performance of cement products. In this study, we have also identified several areas for improvement: The environmental assessment method is relatively simple. Future research should incorporate more factors, such as life-cycle assessment and cost, to conduct a more comprehensive evaluation. Furthermore, the carbon dioxide emission value produced after the calcination of CG at a temperature of 750 °C has not been subject to actual measurement but has rather been directly cited, and this value is relatively small.

## Figures and Tables

**Table 1:** Chemical composition of the materials (wt. %).

Materials\Oxide	CaO	SiO_2_	Al_2_O_3_	Fe_2_O_3_	MgO	Na_2_O	K_2_O	SO_3_	LOl ^y^
OPC	60.20	21.58	5.00	2.11	3.78	0.58	0.13	2.31	4.31
CG	1.48	35.56	58.96	1.43	0.36	0.11	0.26	0.62	1.22
CS	69.0	3.49	1.63	0.34	\	0.53	\	\	25.01

^y^ Loss on ignition.

**Table 2:** Sample mixtures.

	Group	OPC	CS	CCG	Sand	Water	W/B
paste	OPC-100	100	0	0	\	50	0.5
	CS-10	90	10	0	\	50	0.5
	CS-20	80	20	0	\	50	0.5
	CCG-10	90	0	10	\	50	0.5
	CCG-20	80	0	20	\	50	0.5
	CS-10-CCG-10	80	10	10	\	50	0.5
mortar	OPC-100	100	0	0	200	50	0.5
	CS-10	90	10	0	200	50	0.5
	CS-20	80	20	0	200	50	0.5
	CCG-10	90	0	10	200	50	0.5
	CCG-20	80	0	20	200	50	0.5
	CS-10-CCG-10	80	10	10	200	50	0.5

**Table 3:** Summary table of characteristic peaks.

Phase	Ettringite	Calcite	Portlandite	Quartz	Monocarboaluminate
Characteristic peaks	8.95	22.85; 29.45; 46.5	4.70; 18; 29.25; 34.2	26.55; 40.8; 50.8	11.7

**Table 4:** Percentage TGA mass loss (wt.%).

	C-S-H, Aft, AFm, and C-S-A-H	Ca(OH)_2_	CaCO_3_
OPC-100	7.01	3.70	4.70
CS-20	6.89	4.3	4.60
CCG-20	8.22	1.88	5.20
CS-10-CCG-10	8.67	1.59	5.00

**Table 5:** The quantity of each raw material in one cubic meter.

	Group	OPC (kg)	CS (kg)	CCG (kg)	Sand (kg)	Water (kg)
mortar	OPC-100	630.02	0	0	1260.04	315.01
	CS-10	564.12	62.68	0	1253.60	313.40
	CS-20	499.05	124.76	0	1247.62	311.91
	CCG-10	564.34	0	62.71	1254.10	313.53
	CCG-20	499.33	0	124.83	1248.32	312.08
	CS-10-CCG-10	499.23	62.40	62.40	1248.08	312.02

## Data Availability

The original contributions presented in this study are included in the article. Further inquiries can be directed to the corresponding author.
